# Genomic dissection and prediction of heading date in perennial ryegrass

**DOI:** 10.1186/s12864-015-2163-3

**Published:** 2015-11-11

**Authors:** Dario Fè, Fabio Cericola, Stephen Byrne, Ingo Lenk, Bilal Hassan Ashraf, Morten Greve Pedersen, Niels Roulund, Torben Asp, Luc Janss, Christian Sig Jensen, Just Jensen

**Affiliations:** Department of Molecular Biology and Genetics, Center for Quantitative Genetics and Genomics, Aarhus University, Blichers Allé 20, 8830 Tjele, Denmark; Department of Molecular Biology and Genetics, Crop Genetics and Biotechnology, Aarhus University, Forsøgsvej 1, 4200 Slagelse, Denmark; DLF A/S, Research Division, Højerupvej 31, 4660 Store Heddinge, Denmark

**Keywords:** Genomic selection, Perennial ryegrass, Heading, Flowering, GWAS, Lolium, CONSTANS, Phytochrome, Accuracy

## Abstract

**Background:**

Genomic selection (GS) has become a commonly used technology in animal breeding. In crops, it is expected to significantly improve the genetic gains per unit of time. So far, its implementation in plant breeding has been mainly investigated in species farmed as homogeneous varieties. Concerning crops farmed in family pools, only a few theoretical studies are currently available. Here, we test the opportunity to implement GS in breeding of perennial ryegrass, using real data from a forage breeding program. Heading date was chosen as a model trait, due to its high heritability and ease of assessment. Genome Wide Association analysis was performed to uncover the genetic architecture of the trait. Then, Genomic Prediction (GP) models were tested and prediction accuracy was compared to the one obtained in traditional Marker Assisted Selection (MAS) methods.

**Results:**

Several markers were significantly associated with heading date, some locating within or proximal to genes with a well-established role in floral regulation. GP models gave very high accuracies, which were significantly better than those obtained through traditional MAS. Accuracies were higher when predictions were made from related families and from larger training populations, whereas predicting from unrelated families caused the variance of the estimated breeding values to be biased downwards.

**Conclusions:**

We have demonstrated that there are good perspectives for GS implementation in perennial ryegrass breeding, and that problems resulting from low linkage disequilibrium (LD) can be reduced by the presence of structure and related families in the breeding population. While comprehensive Genome Wide Association analysis is difficult in species with extremely low LD, we did identify variants proximal to genes with a known role in flowering time (e.g. CONSTANS and Phytochrome C).

**Electronic supplementary material:**

The online version of this article (doi:10.1186/s12864-015-2163-3) contains supplementary material, which is available to authorized users.

## Background

Perennial ryegrass (*Lolium perenne* L.) is one of the most cultivated forage species in temperate grasslands, mainly farmed for its re-growth capacity after defoliation, and for the high value as feed for ruminants, due to palatability, digestibility, and nutritive contents [1–3]. Perennial ryegrass is an obligate allogamous species with genetic gametophytic self-incompatibility [4], and is bred in genetically heterogeneous families.

Heading date (HD) is an important trait for forage species, often used as a model trait [5, 6] due to its high heritability and the ease of assess. It follows the shift from vegetative to reproductive growth, and it is significantly correlated with several other traits involved in plant growth and development, such as plant height, spike length, tiller number and size, leaf length [7, 8], as well as with a number of yield and quality traits. Early heading genotypes show a higher growth rate in spring and higher forage yield in the first cut, [9–12]. Correspondingly, the opposite was found for later cuts in summer, where dry matter forage yield was higher for late genotypes. Results in the literature are inconsistent regarding performances in fall and winter. Humphreys [9] found higher autumn and winter growth rate in late genotypes, while in Sampoux et al. [11], the correlation between HD and forage yield in autumn was not significantly different from zero. Differences between early and late genotypes were also observed in the intensity of aftermath heading, which was higher in the early material [9, 11, 12], as well as in the content of fiber and soluble sugars. Humphreys [9] and Sampoux et al. [11] measured less water soluble carbohydrates and more lignin and neutral detergent fiber in early- than in late HD varieties. Late heading was also associated with higher digestibility and therefore to a higher lactation energy content for milk production [10]. Although heading also marks the production of seeds, the correlation with seed yield is unclear. Later genotypes were generally found to give lower seed production [13, 14], but this correlation was not always significant [8, 14].

Due to its significant effects on other traits, breeding has always aimed to exploit the natural variation in HD, in order to create mixtures of varieties that could give high performances throughout the whole year. Since International listing of new varieties requires fulfilment of the three criteria; distinctiveness, uniformity, and stability (DUS) there is also a strong breeding focus on HD in order to create uniform varieties. While the uniformity of inbred varieties is rather easy to control it can be more challenging in outbreeding grass varieties that are breed as families.

In order to ensure more stability in forage quality over the season, cultivars have been divided into different earliness groups. The number- and extend of each HD group differs between countries, with some countries defining up to nine HD groups. However, HD appears to behave as a continuous character, and the distinction between early and late material is not always clear, with new candidates that may be classified in different neighboring HD groups, depending on the definitions used in the different countries. The trait always showed medium to high heritability [9, 12, 14–16]. Kearsey et al. [15] showed the presence of both additive and dominance effects, with the first being the larger and dominance being for early heading, but did not find any evidence for epistasis. Genotype by environment (G × E) interactions were found to be small by Ravel and Charmet [16], in a multi-site analysis in France. However, a different result was obtained by Kearsey et al. [15], who showed interactions between the environment and both additive and dominance effects, in an experiment across Italy and the UK.

In the latter decade, the genetic control of HD was better understood thanks to the use of molecular markers and comparative genome analyzes. In model species, such as *Arabidopsis thaliana* L., as well as in cereals like wheat and rice heading or the control of flowering has been the subject for numerous studies and publications (reviewed in [17–19]). Especially the use of induced Arabidopsis mutants and the combinations of such lead to the detailed modelling of the genetic control of flowering in plants. The investigations demonstrated the involvement of genes belonging to three major pathways: (i) vernalization response genes (*Vrn*), which regulates heading after low temperature periods; (ii) photoperiod response genes (*Ppd*), which is active/inactive with a certain day length; (iii) ‘earliness per se’ factors, which seems to be independent of light and cold requirements [20].

In perennial ryegrass a number of flowering genes were previously identified by sequence homology with flowering genes found in Arabidopsis, rice, and maize [17, 21–23]. Others were identified through classical Quantitative Trait Loci (QTL) mapping, performed using different plant material and different genetic maps. Genetic maps were organized in seven linkage groups (LGs), numbered according to the conserved synteny with the *Triticeae*’s maps [24]. QTLs were identified on all seven LGs [7, 8, 25–29]. Comparison between studies is complicated due to lack of common markers and it is always difficult to determine if two significant markers found on the same LG, actually correspond to the same QTL. Furthermore, among different studies there is often poor agreement regarding the number and the distribution of the QTLs, likely due to environmental factors, use of different mapping populations [30], and low statistical power in several studies. A great effort was put in understanding the genetic mechanisms behind the QTLs in LG4 and LG7, which were significant in almost all studies. The first was found to be in a syntenic association with the wheat *Vrn1* gene [26], and its function seems to be conserved between diploid wheat and perennial ryegrass [19]. A relation was also hypothesized with a putative casein kinase gene, previously mapped in rice and involved in photoperiod sensitivity [29]. The QTL on LG7 was suggested to be associated with the gene *LpCO*, homologous to the *CONSTANS* of *Arabidopsis* and the *Hd1* of rice, involved in the photoperiodic regulation of flowering time [17, 31, 32]. Synteny was also detected with the *Hd3* region of rice [25], which codes for a *FLOWERING-LOCUS-T* (*FT*) orthologue of Arabidopsis. *FT* gene is involved in induction to reproductive growth at the meristem [33, 34] and has been shown to actively regulate the flowering response in *L. perenne* [35]. Other hypothesis have been proposed to relate the other significant markers to QTL previously found in related species, such as *Lolium multiforum* Lam. and *Festuca pratensis* L. [29].

While these studies identified some of the key genes in floral control in ryegrass, the biology of the trait is still far from being understood. Furthermore, the use of QTL analyses was shown to be not effective in capturing small effect genes [36] and to overestimate the variance explained by QTLs, due to the so called Beavis effect [37, 38]. However, such limitations may be overcome by the use of Genomic Selection (GS). In contrast to traditional Marker Assisted selection (MAS), GS does not focus on finding specific QTLs, but selects families/individuals based on Genomic Estimated Breeding Values (GEBV), which are calculated using all markers simultaneously. Linkage Disequilibrium (LD) between causative loci and markers is ensured by high marker coverage. Such LD can come from three sources: (i) close physical linkage between marker and QTL; (ii) family structure in the population, creating both short range (within chromosome) and long range (across chromosomes) LD; (iii) population structure due to mixing breeding material of different origin. Therefore, the LD can be also tracked across families, enabling to estimate marker effects at a population level [36].

GS is practically implemented trough different steps: (i) model development on a set of individuals/families that are both genotyped and phenotyped (training set); (ii) estimation of GEBVs for a set of individuals/families that are only genotyped (validation set), based on their relationship with the training set; (iii) selection of the best breeding material. In this paper we will refer to the first two steps as Genomic Prediction (GP). GS is now widely used in animal breeding [39], but it is still a new technology in crop breeding. To date only a limited number of studies has been published on real data, mainly on species that are primarily grown as homogeneous varieties, such as maize, barley, and wheat (reviewed in [40]). The first results are promising and GS is expected to significantly increase genetic gains, especially due to the shortening of the breeding cycles [41]. So far, aside from a few theoretical discussions, very little has been reported about GS potentials in allogamous species that are breed and farmed as heterogeneous populations. Specifically for perennial ryegrasses, Hayes et al. [42] showed good perspectives for introducing GS in practical breeding programs. However, a full implementation would require radical changes in the present breeding systems, and may face problems due to low LD and high effective population size, due to the outcrossing nature of the species [42].

This paper represents our first attempt to introduce GP in a breeding program of forage perennial ryegrass, using HD as model trait. 1757 F_2_ families (F_2_s), phenotyped for HD and genotyped with high marker coverage, were used to dissect the genetic and genomic structure of the trait. First, a Genome Wide Association Analysis (GWAS), to check for the presence of major QTL was conducted. Second, significant markers were used to calculate the GEBVs in a set of synthetic (SYN) families, a part of which was related with the training set. Third, GP models were tested within the F_2_ set, using different cross-validation (CV) schemes and different population sizes, and then used to predict breeding values of SYN families. Predictive ability of GP was compared with predictions based on GWAS results.

## Results

### Population structure, LD, and genetic parameters

**Fig. 1:**
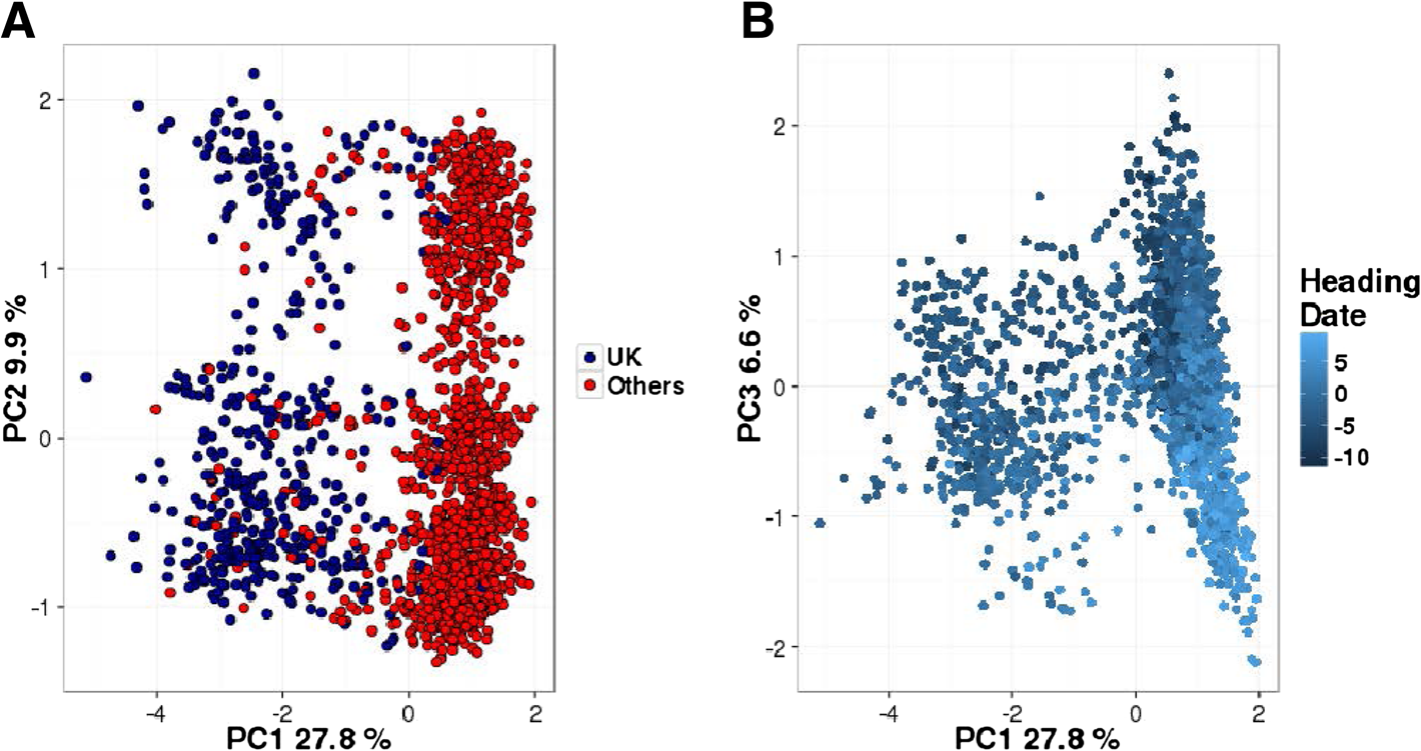
First PCs and: **a** origin of the PPs; **b** breeding value for HD

Results from the Principal Component Analysis (PCA) showed the presence of some degree of population structure (Fig. 1). The ‘elbow’ point of the PCA scree plot was determined at the fourth PC (Additional file 1: Figure S1). The first four PCs explained 28, 10, 7, and 6% of the variance among SNPs respectively. The optimal numbers of cluster, determined by k-means clustering, turned out to be two. The separation in the two clusters could be explained by the first PC and it was strongly related to the origin of the Parent Populations (PPs). In Fig. 1a, all the families represented by blue points were identified as pair-crosses having a varieties originated in UK as one PP. For this reason, in the following part of the paper, we will refer to this group as (UK). The population structure was also shown to be related to HD, which was mostly explained by the third PC (Fig. 1b).

**Fig. 2:**
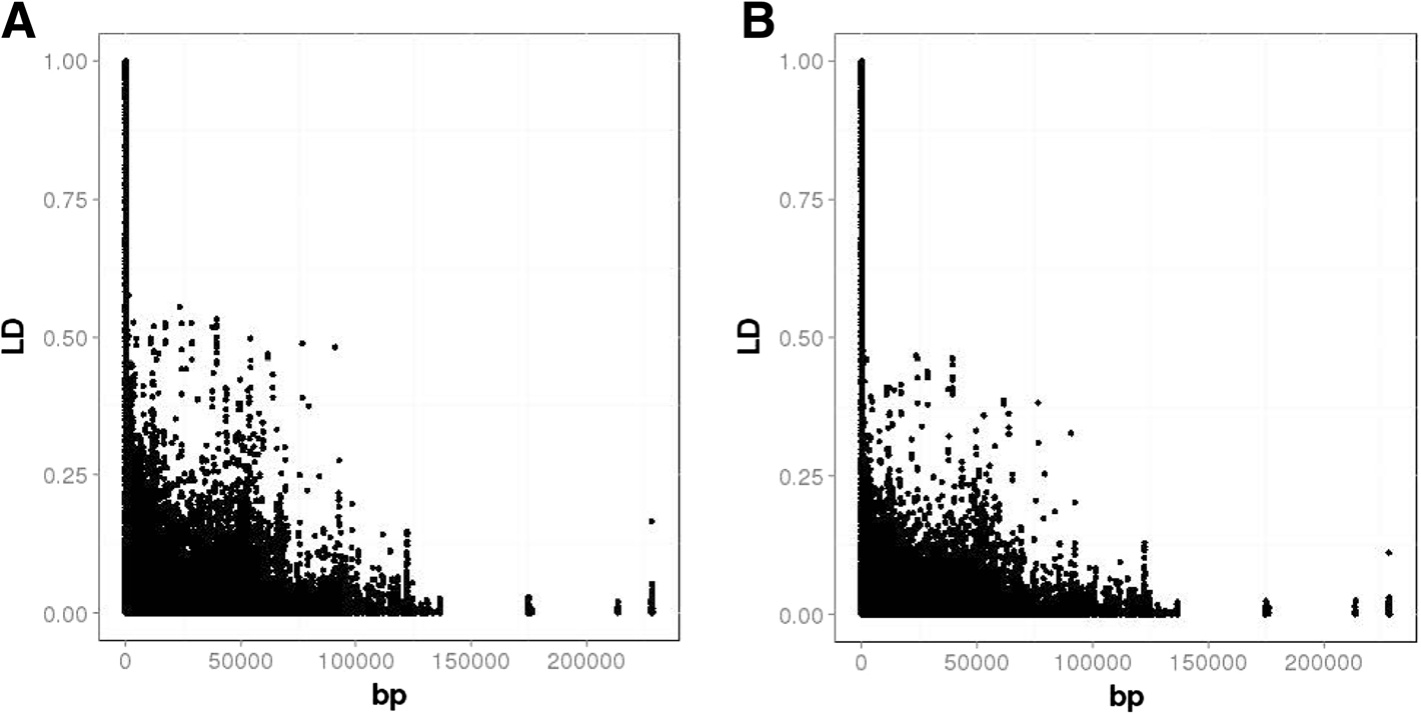
LD decay: **a** without corrections; **b** corrected for relatedness and population structure

Results from the LD analysis are displayed in Fig. 2. The LD was shown to have a rather short extent, decaying below 0.5 after a few hundred base pairs (bp). Without using any correction for relatedness and population structure (Fig. 2a), the markers with LD > 0.10 and LD > 0.25 were the 6.3 % and the 3.37 % of the total number of SNPs respectively. The average distance between markers having LD > 0.10 was about 8900 bp, and for markers having LD > 0.25 the distance was close to 3600 bp. The correction further reduced the proportion of SNPs in LD (Fig. 2b), which dropped down to 3.4 % for LD > 0.10 and to 1.43 % for LD > 0.25, corresponding to a reduction of 46 and 40 % respectively. The average distance between markers having LD > 0.10 was reduced to 6300, while the one for markers having LD > 0.25, drop down to 1200 bp, corresponding to a reduction of 29 and 66 % respectively, showing that the correction for structure and relatedness reduced the short range LD in the population. The proportions of SNPs separated by more than 1200 and 6300 bp were about 86 and 71 % respectively.

**Fig. 3:**
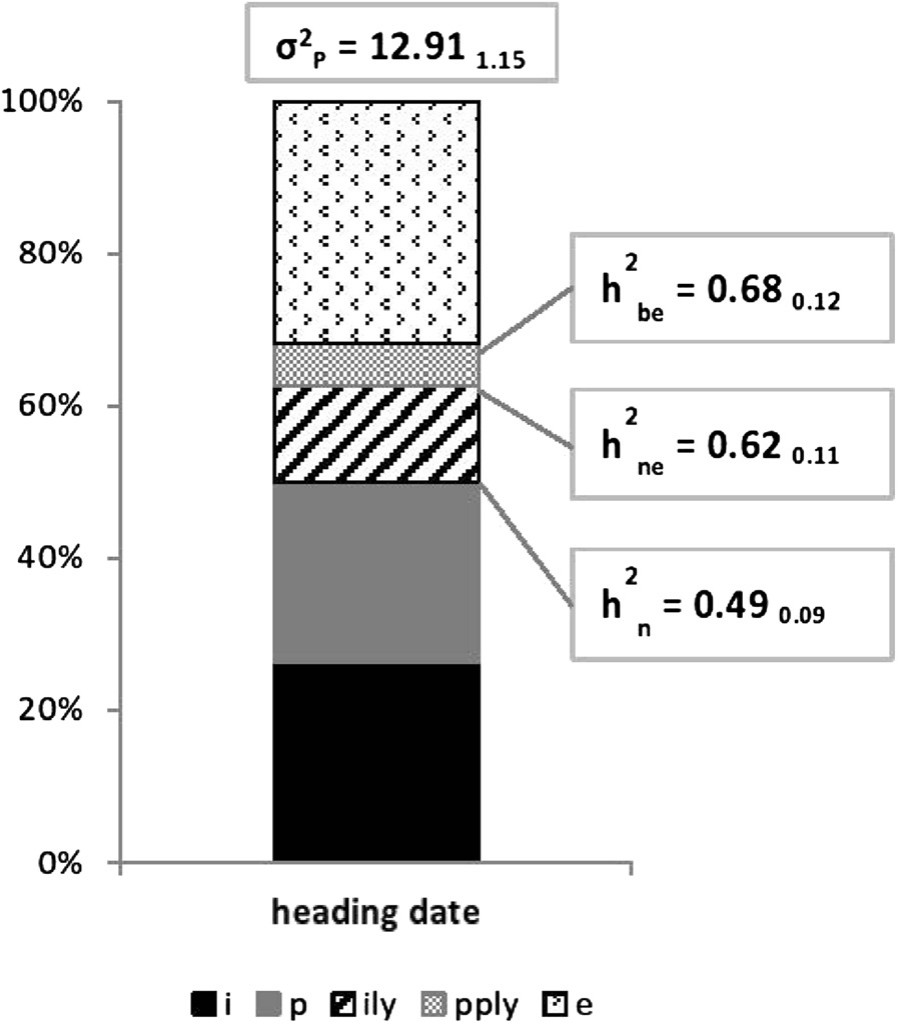
Variance components, phenotypic variance, and heritabilities (with SE). Legend: i = **σ** ^**2**^_**i**_; p = **σ** ^**2**^_**p**_; ily = **σ** ^**2**^_**ily**_; pply = **σ** ^**2**^_**pply**_; e = **σ** ^**2**^_**e**_; **h** ^**2**^_**n**_ = narrow sense heritability across environments; **h** ^**2**^_**ne**_ = narrow sense environment-specific heritability; **h** ^**2**^_**be**_ broad sense environment-specific heritability

The total amount of phenotypic variance, together with different definition of heritability, is shown in Fig. 3. The additive genetic variance accounted for half of the total phenotypic variance, and it was equally divided between the ‘within PPs’ and the ‘among PPs’ components. The interaction between the additive effect and the environment was relatively small (accounting for the 13 % of the total phenotypic variance) and occurred only within PPs.

### Genome wide association

**Fig. 4:**
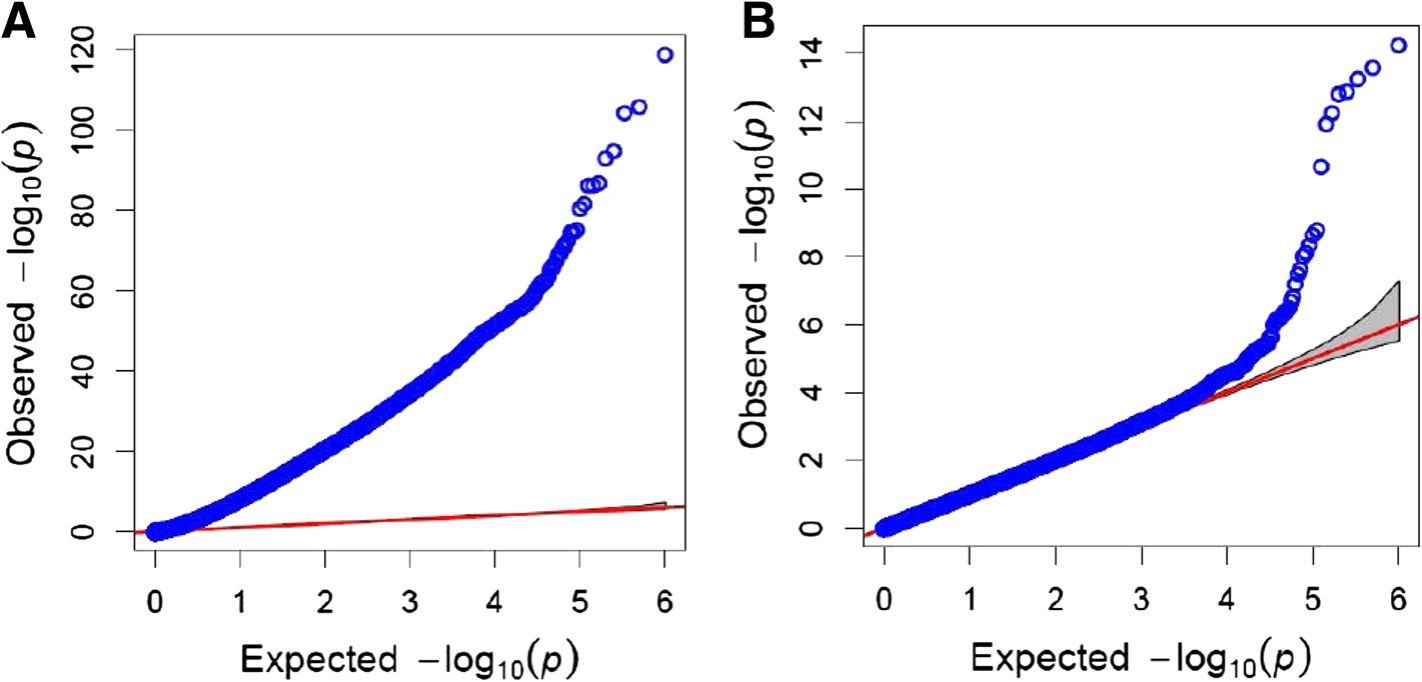
QQ-plots, without (**a**) and with (**b**) correction for G-matrix and PCs

Using the Bayesian Information Criterion, the optimal number of PCs for population structure correction was determined to be four, confirming the visual identification of the ‘elbow’ point. The effect of the correction on the significance levels expressed as –log_10_(P) is clear from the QQ-plots reported in Fig. 4. After selection for high LD within scaffold, the number of significant SNPs (*P* < 0.05) was 10 using the t-test with Bonferroni correction, and 19 using False Discovery Rate (FDR) (Table 1). SNPs are anchored to genomic scaffolds, which are not orientated or ordered with respect to a genetic map. However, the draft assembly has been annotated with the aid of extensive transcriptome data and a number of genes have been predicted in the scaffolds harboring the significant SNPs (Additional file 2 and Additional file 3: Table S1 in the supplementary material). A total of ten markers were found to be within the gene space, 9 of which were mapped in exon regions (Table 1). The allele substitution effects ranged from 0.40 to 1.39 days. The percentage of additive variance across locations/years explained by each marker was between 0.59 and 1.82 % within the F_2_ families, and between 0.28 and 1.06 % in the SYN families. The sum of the variances explained by all significant markers corresponded to about 20.3 % in the F_2_s and 11.2 % in the SYNs. The correlation between the marker effect in the two sets was positive (r^2^ = 0.22).

**Table 1:** Summary statistics for all the significant SNPs

Scaffold|Position	Location	MAF	α	% σ^2^_g_(F_2_)	% σ^2^_g_(SYN)	*P*-value Bonferroni	*P*-value FDR
3546|38401	outside gene	0.08	1.19	1.62	0.65	6E-09	6E-09
18961|1999	exon	0.12	1.00	1.70	0.74	3E–08	1E–08
18961|3412	exon	0.27	0.57	1.01	0.71	0.004	4E–04
6570|54193	outside gene	0.11	1.03	1.68	1.02	6E–07	9E–08
22974|3466	outside gene	0.06	1.39	1.82	1.06	1E–06	2E–07
22974|2499	outside gene	0.22	0.74	1.47	0.86	2E–05	3E–06
1379|64623	outside gene	0.09	0.84	0.92	0.33	0.002	2E–04
1379|60655	exon	0.23	0.56	0.87	0.53	0.147	0.009
18588|6786	intron	0.06	1.14	1.19	0.37	0.002	2E–04
18588|6657	exon	0.28	0.50	0.79	0.52	0.417	0.020
18588|6882	exon	0.06	1.01	0.98	0.45	0.696	0.027
9291|22927	outside gene	0.18	0.59	0.80	0.46	0.007	6E–04
9679|461	outside gene	0.20	0.59	0.88	0.41	0.010	7E–04
2801|42855	exon	0.33	0.51	0.91	0.56	0.355	0.018
5059|6359	exon	0.25	0.51	0.77	0.53	0.457	0.021
3169|35325	exon	0.06	1.05	0.90	0.75	0.503	0.022
21110|2619	outside gene	0.17	0.52	0.59	0.28	0.597	0.025
3586|39964	exon	0.43	0.40	0.61	0.43	0.730	0.027
3395|30371	outside gene	0.39	0.47	0.82	0.54	0.837	0.030

The SNP 5059|6359 is situated in a scaffold where the *Hd1* homolog of the *LpCO* gene was also mapped. The marker 2801|42855 locates in a gene encoding for *Phytocrome C* (*PHYC*). The scaffold 1379 harbored two significant SNPs: 1379|60655, situated in a gene encoding for a *Pectate Lyase 4*, and 1379|64623, located outside the gene space, but only 3500 bp away from the *Pectate Lyase 4* coding region.

**Fig. 5:**
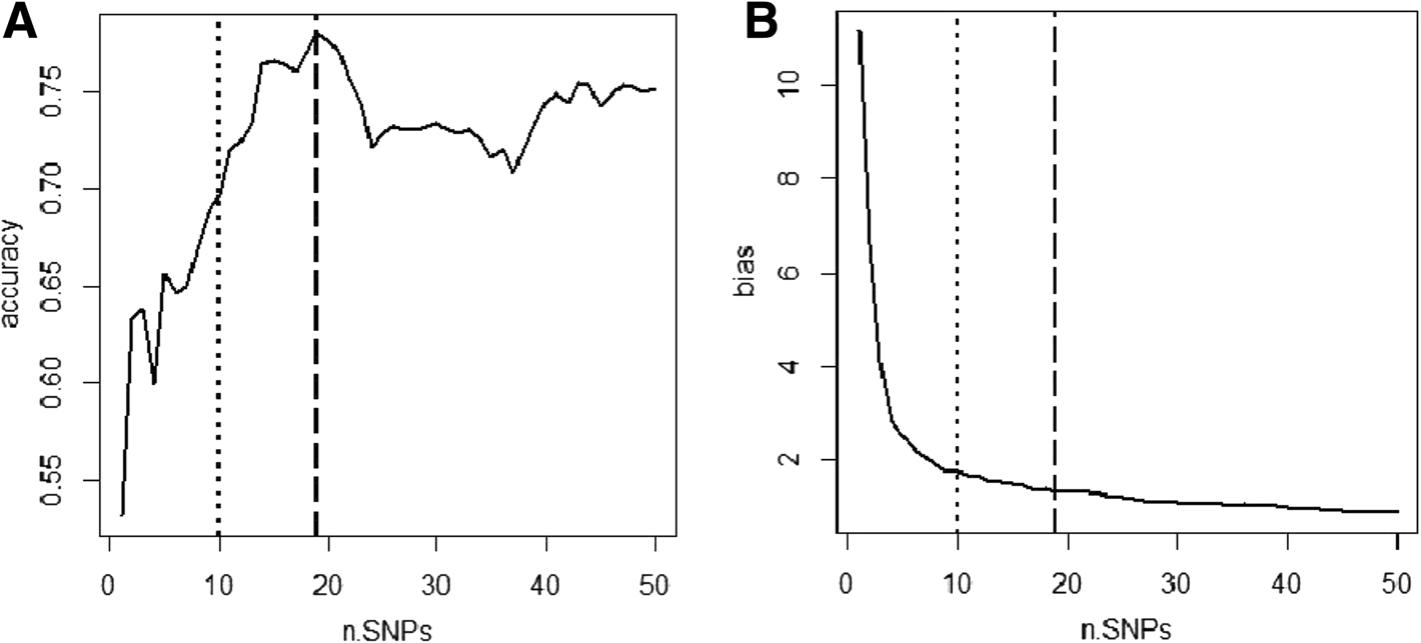
Accuracies (**a**) and bias (**b**) for prediction of SYNs, based on marker effects. Legend: the vertical lines indicate the two significant thresholds: Bonferroni corrected t-test (dotted line), and FDR (dashed line)

Prediction of SYNs based on SNP markers is shown in Fig. 5. Using only the most significant SNP, it was possible to get an accuracy of predictive ability of 0.53. Adding more markers, initially improved the predictions. Accuracy was 0.70 using all the markers that were declared significant with the Bonferroni corrected t-test. The highest correlation between real and estimated breeding values was reached by using all markers that passed the significance threshold in the FDR test. The estimate was equal to 0.78, meaning that all significant SNPs were able to predict the 60.8 % of the genetic variance in the SYN families. Keeping adding markers after that threshold did not lead to any improvement in the predictions, giving accuracies that kept fluctuating between 0.69 and 0.78. Concerning the bias of the estimates, using only a few markers clearly led to a downward bias in the variance of the predicted breeding values. The underestimation was on the order of 2 when the first 10 SNPs were used. Adding more markers led to a significant reduction in bias. At the point of maximum predictive ability, it was 1.33, and it kept decreasing even if insignificant markers were added.

### Genomic prediction

**Fig. 6:**
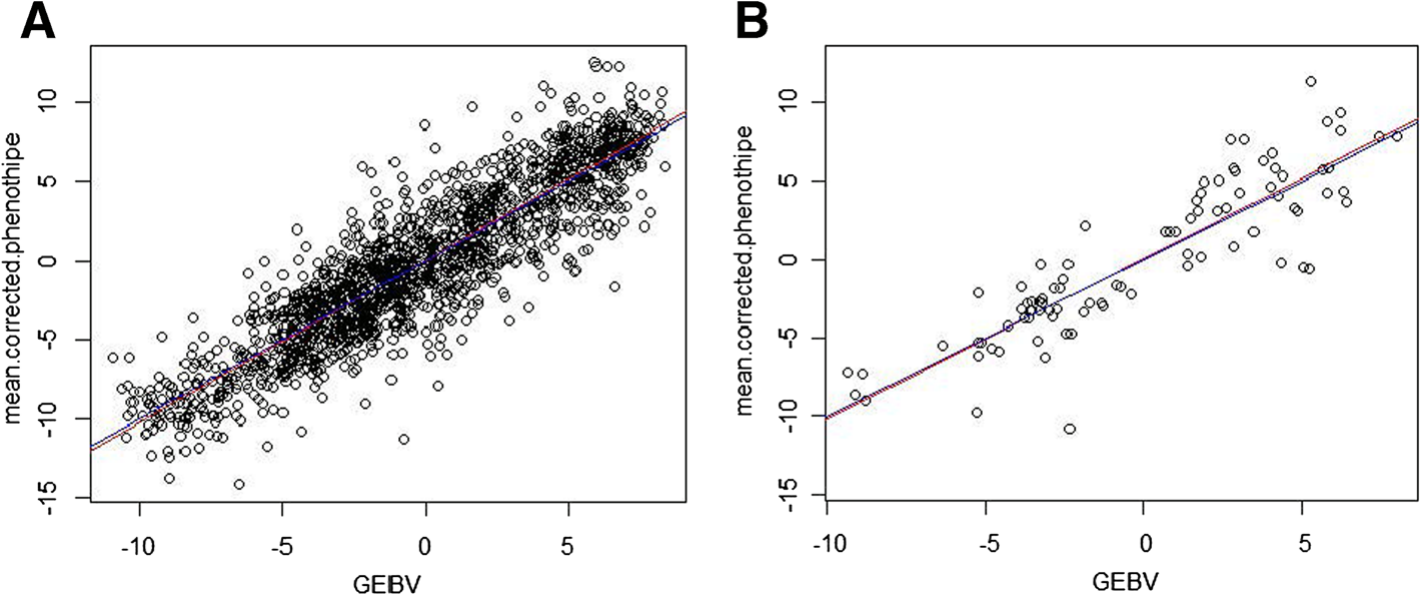
GEBV vs. corrected mean phenotypes: **a** within F_2_s (*k-fold*); **b** predicting SYNs from F_2_s. Legend: blue line = plot diagonal; red line = linear regression

**Fig. 7:**
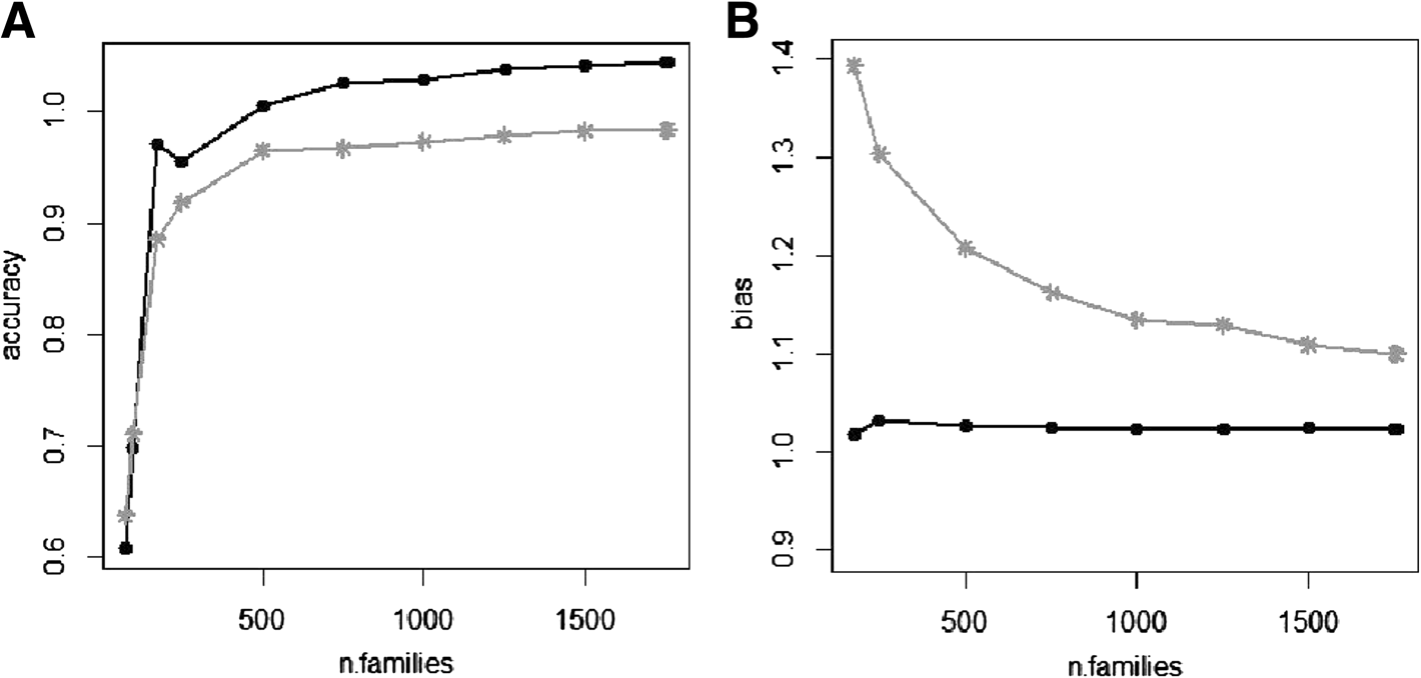
Accuracies (**a**) and bias (**b**) with different population sizes: *k-fold* (black) and *pp-fold* scheme (grey)

For each Cross-validation (CV) scheme, predictive abilities, accuracies, and bias are displayed in Table 2. Within F_2_s, predictive abilities were extremely high, reaching the value of 0.84 in the absence of related families in the two sets (*pp-fold* scheme), and 0.90 when training and validation set contained related families, in the so called *k-fold* scheme (Fig. 6a). The Hotelling-Williams test showed the two values to be significantly different (*P*<0.001). For the *k-fold* scheme, the accuracy computed using the approximation in (14) gave an estimate that was higher than one (1.04). However, the standard error (SE) of this estimate was 0.07, indicating that the actual accuracy could range between 0.91 and 1.00. Analyses on reduced training sets (Fig. 7) showed that accuracies above 0.95 were reachable, for both schemes, with training populations as larger than 500 families. Bias was shown to be always very low in the *k-fold* scheme. The situation was different in the *pp-fold* CV, where the GEBVs variance was generally underestimated, and where an increase in the population size resulted in a bias reduction: the regression coefficient (*b*) was 1.39 using 175 families, and 1.10 using the full dataset. Bias for population sizes below 175 families is not shown, because affected by very large SE and not indicative of any trend. Accuracies within the set (UK) were lower than the ones found on an equal number of randomly chosen F_2_s, especially in the *pp-fold* scheme. Bias was also slightly higher. The CV within the other cluster gave more or less the same results as the CV within all F_2_s.

**Table 2:** Population size and results (with SE) for all CV schemes

CV scheme	Pop.size	ρ_ӯf;ĝ_^†^	ρ_g;ĝ_	bias (*b*)
k-fold	1757	0.90 _0.01_^a^	1.04 _0.07_	1.02 _0.01_
pp-fold	1757	0.84 _0.01_^b^	0.98 _0.06_	1.10 _0.02_
k-fold (UK)	466	0.78 _0.03_^a^	0.86 _0.09_	1.06 _0.04_
pp-fold (UK)	466	0.52 _0.04_^b^	0.57 _0.07_	1.30 _0.10_
k-fold (others)	1291	0.90 _0.01_^a^	1.04 _0.07_	1.02 _0.01_
pp-fold (others)	1291	0.86 _0.01_^b^	0.99 _0.07_	1.17 _0.02_
UK - > others	466	0.78 _0.02_^N^	0.90 _0.07_	1.46 _0.03_
Others - > UK	1291	0.71 _0.03_^N^	0.78 _0.08_	0.92 _0.04_
F2s - > SYNs (GS)	1757	0.88 _0.05_^a^	0.93 _0.24_	1.02 _0.06_
F2s - > SYNs (GWAS)^‡^	1757	0.74 _0.07_^b^	0.78 _0.21_	1.33 _0.13_

^†^different letters indicate a significant difference between the two CV schemes (*P* < 0.001) based on Hotelling-Williams test. N indicates that the comparison does not apply, as models were based on different sets of data

^‡^using all SNPs that were declared significant after FDR test

Predictions across sets also worked well. Accuracy of predicting UK set from the other F_2_ families was slightly lower (accuracy equal to 0.78). Predictions for GEBVs were better when the set (UK) was used as training set. In this case, accuracies were comparable to the ones obtained within all F_2_s, with a *pp-fold* scheme, and using a similar population size. The bias level indicates that the GEBVs variance was underestimated when the set (UK) was used as training population, and slightly overestimated when the prediction was performed in the opposite direction. Predictive ability for SYNs (Fig. 6b) was similar to the ones within all F_2_s, and significantly different from the one obtained from GWAS results (*P*< 0.001). The accuracy was 0.93, 14 % higher than the in prediction based on the significant markers. In this case, the linear regression of mean corrected phenotypes on GEBVs indicated no bias in the GEBVs variance.

## Discussion

### Population structure, LD, and genetic parameters

The population structure was mainly defined by the origin of the PPs, which was correlated with the first principal component. The majority of the F_2_ families were grouped in one big cluster. This may lead to the hypothesis of a common European genetic pool. This pool is likely to originate from the continuous and (more or less) free exchange of breeding material among the different breeders. The parents of the set (UK) may be an exception to that pool, and their genetic origin need to be further investigated. The relation between PC3 and HD indicates the need to correct for population structure while performing GWAS, in order to avoid false positives. Further variance analyses were performed by adding fixed regressions for the first 1, 2, 3, and 4 PCs to the equation shown in formula (2). Result indicates a correlation of HD with the PC3, but not with the other three main PCs. When accounting for the first two PCs, the additive genomic variance across location was equal to the 98 % of the ones of the model without any PC. When accounting for the first three PCs, the additive genetic variance left was the 89 %. Adding a regression for PC4 had a negligible effect.

The LD within scaffolds showed to decay rapidly, confirming the concerns expressed by Hayes et al. [42], who reported useful LD (r^2^ > 0.25) to extend at best 1 kb. However, in the present breeding material, the presence of relatedness and population structure generally increased the LD, bringing to an increase by the order of three in the average distance between those markers. This fact suggests that population structure, which is known to be mostly responsible for the long range LD, also plays an important role in increasing the level of LD within scaffolds. The correction also led to a decrease in the average distance between markers in LD, which was more pronounced for higher LD levels.

Estimation of variance components confirm results obtained by Fè et al. [12] on a subset of the same data. In this paper was also possible to calculate the heritability across environments, and to estimate the extent of G × E for additive and non-additive effects. Compared with other traits previously analyzed [12], the proportion of genetic variance between PPs was much higher. The small level of G × E seems to confirm the results obtained by Ravel and Charmet [16]. However, plants were cultivated only in Denmark and England. To have a better understanding of G × E effects, it would be a good idea to perform experiments covering more diverse climatic conditions. The significant amount of **σ**^**2**^_**pply**_ may indicate the presence of dominance acting between families and within single environments. In the literature, the presence of non-additive effect is reported also across location [15]. However results are not directly comparable, as this paper ignores additive effects that may be present within F_2_ families.

### Significant markers and genetic architecture

The GWAS analysis revealed a rather complex genetic architecture of HD in ryegrass. Several markers with significant effect were identified. In the most significant SNPs, a shift from one homozygous form to the other can cause changes of up to 2.78 days in the date of heading. That is a remarkable difference, if compared with the level of variation in the phenotypes: average phenotypes corrected for fixed effect had a SD of 4.92. However, due to low Minor Allele Frequency (MAF), these markers were only able to explain a small proportion of the additive variance, which may indicate the presence of a large number of genes also affecting the trait, but with effects lower than the detection limit. There were a high number of significant SNPs found outside the gene space. This is not too surprising considering a recent study in maize found that the majority of trait associated variants were located outside annotated genes, but within 5 Kb of transcriptional start and stop sites [43].

Some of the significant SNPs were clearly linked to genes that may have a direct or indirect influence on HD. It is well established that *CONSTANS* (*CO*) plays a crucial role in promoting flowering in response to long days [44]. We identified a significant SNP within less than 5 Kb of a *CO* homolog. It has already been established that a ryegrass homolog to *CO* exhibits expression patterns consistent with its function in Arabidopsis, and can complement co mutants [17]. Furthermore, the *CO* homolog co-located on linkage group seven with a large effect QTL for HD [19, 31]. Allelic variation in an intergenic region upstream of *CO* was found to be significantly associated with HD in a collection of 96 perennial ryegrass genotypes (originating from nine populations) [32]. The ~29 Kb region sequenced as part of that study shares near perfect identity with scaffold 5059 (Additional file 4: Figure S2), which has the significant SNP identified in our study, and therefore represent the same genomic regions. Overall, our results provide further evidence that allelic variation at *CO* contributes to variation in HD in perennial ryegrass, specifically within a large collection of breeding families.

We also identified a significant SNP within the coding region of a homolog to *PHYC*. Phytochromes are red/far-red photoreceptors that play a role in how a plant responds to light, and adapts its growth and development. It was recently demonstrated in wheat that *PHYC* plays a major role in accelerating flowering under long-days [45], in contrast to the model plants such as Arabidopsis and rice where *PHYC* represses flowering under non-inductive conditions. The fact that loss-of-function mutations in *PHYC* resulted on average in a 108 day delay in flowering of wheat under long days emphasizes the potential for allelic variation at this gene to greatly alter flowering times. A similar role for *PHYC* has also been recently reported in *Brachypodium distachyon* [46]. Perennial ryegrass is a close relative of wheat and *Brachypodium,* and a similar role for *PHYC* in floral induction of perennial ryegrass is possible. A homolog to *PHYC* has been mapped to linkage group four of perennial ryegrass [47], although it mapped some distance from the HD QTL identified in that experimental population. No correlation was found between significant markers and other genes that are known to be important in flowering time regulation, such as *FT*. That may be due to different reasons: (i) absence of causative polymorphisms in the breeding material; (ii) no or low LD between markers and the causative polymorphisms (likely to happen, due to the fast decaying LD); (iii) low MAF at the causative polymorphisms (about 45 % of the markers had MAF lower than 0.05); (iv) polymorphisms not detected because they are correlated with the family structure and shrunken by the correction with G-matrix and PCs (the third PC was clearly correlated with HD).

### Prediction of breeding values

Despite explaining only a small part of the genetic variance in the SYNs, the significant markers were able to predict the breeding values with high accuracy, even when only a few markers were used. This is due to few genes with relatively large effects identified in the F_2_ population. However, the presence of a certain level of population structure (displayed in Fig. 1) will also contribute to the predictive ability in the SYNs. In the GWAS, we accounted for the presence of population structure by correcting the marker effect (using the G-matrix and the first four PCs). However, that correction does not apply to the estimation of prediction accuracy. When we correlate the phenotypes with one marker, we are actually estimating the correlation of the phenotype with that particular marker, plus all the population structure that is correlated to the SNP. The trend in accuracy for an increasing number of markers met our expectations: any significant SNP is supposed to add information that will increase the correlation with the true breeding value. Non-significant SNPs will mainly add random noise to the correlation, but were able to add genetic information that increased the variance of estimated breeding values. The fact that the accuracy reaches the highest value in correspondence of the nineteenth SNP is also a strong argument for using FDR, instead of Bonferroni corrected t-test, as significance test. The decreases in prediction accuracy that happened after adding the fourth and the sixth markers may be related to different levels of expression or to different interactions in the two populations.

Results from SYNs prediction (Table 2) show a clear advantage for using GP, compared with GWAS, both in terms of accuracies, and in terms of bias, as well as its good potential in predicting across different generations. The relatively high SE for accuracies may be related to the experimental design that, due to incomplete randomization between trials and PPs, which could lead to less accurate estimate of PP variance components. Within all F_2_ families, predictions were extremely good, allowing the explanation of nearly the whole genetic variance. This result is higher than what usually found for the same trait in other species (reviewed in [40]), even though accuracies above 0.8 have already been reported in other outcrossing species such as maize. That may be related with the high level of structure in the population, and to the fact that heading date primarily is affected by additive genetic effects, so the additive values of the PPs are very well estimated.

A very high accuracy may also seem in contrast with what reported in the literature for traits affected by major effect SNPs [48]. Theoretically, for traits that include some genes with large effect, it would be recommended to use other prediction methods such as Bayesian models, which allow marker effects to belong to distributions with different variance. However, Genomic Best Linear Unbiased Prediction (GBLUP), when compared with Bayesian methods, was shown to be better in accounting for population structure, but less capable to explain the short range LD between markers [36]. This makes it particularly effective for GP in breeding programs of species like perennial ryegrass, characterized quick decay of short range LD, and usually bred on a sib-mating scheme. The lower accuracies found within the set (UK) may be related to a low level of population structure within the cluster, as appear also from Fig. 1.

Accuracies reached by predicting from related families (*k-fold* scheme) were significantly higher than the ones obtained in the absence of related families (*pp-fold* scheme), for any population size. Regarding the relationship between predictions and the size of training population, increasing the training size led to pronounced gains in predictive abilities for population sizes lower than 500 families, and to smaller gains in the case of larger populations. Despite that, due to the higher predictive abilities, accurate predictions could be obtained even using a relatively small training set. Problems of underestimations of the GEBVs variance may arise in absence of closely related families. Regarding predictions across sets, the relative difficulty in predicting the set (UK) may be due to families in this set having fewer relatives in the chosen training population. The level of bias may arise due to the differences in genetic variance or to genetic correlations that are less than unity between the two sets. This problem could compromise a correct comparisons between GEBVs from the training set (which have known bias) and the validation set (which have unknown bias). This should be taken into consideration during the design of the training population, allowing the presence a wide variety of genotypes in as many environments as possible.

## Conclusions

Our research clearly showed considerable potential for implementation of GS in breeding of *L. perenne*. Results obtained by GP significantly outperformed the accuracy based on traditional MAS, being able to predict a very large proportion of the genetic variance. GBLUP was shown to be capable of reaching very high accuracies, even in a trait characterized by major effect genes, at least in a population with fast decaying LD and population structure arising from admixture and relatedness. Predictions were also very good across datasets, with accuracies of up to 0.93. Bias in the GEBV variance could be caused by lack of common parent populations between training and validation set.

The study has also revealed important details about the genetic architecture of HD in *L. perenne*. The trait appears to be controlled by both major and minor effect genes, regulated both by sequence changes within coding regions, and by the action of intergenic regulatory elements. SNPs were identified within or proximal to genes with well-established roles in floral induction in plants (*CONSTANS* and *PHYC*). Despite this, the technique used for GWAS has limitations, mainly due to the marker density given the rapid decay of LD, and due to the strong structure in the population.

## Methods

### Plant material, genomic and phenotypic data

Both Phenotypic and genomic data were available for a total of 1846 families of forage diploid perennial ryegrass. All breeding material was part of a standard forage breeding program run by DLF A/S (Store Heddinge, Denmark). Unlike cereal breeding, population based forage breeding usually does not advance further than the second generation. Each year, the best breeding material is selected and added to the company’s gene bank, which also includes European varieties, commercialized both by DLF and other companies.

The plant material consisted of two different sets:

- Set 1. F_2_s: 1757 F_2_ families produced across 13 years (between 2000 and 2012) from a seed bank of 198 PPs. Development of F_2_ families was detailed in Fè et al. [12]. In brief: (i) pair-crosses between single plants from two different PPs (self-pollination avoided due to self-incompatibility). Each single plant was used only in one pair-cross; (ii) seed harvesting from both parent plants; (iii) pooling of the F_1_ seeds; (iv) isolated multiplication of F_1_ populations in isolated plots for random mating; (v) harvesting of F_2_ seeds; (vi) field trials of F2 families (assumed to be in Handy-Weinberg equilibrium).
- Set 2. SYNs: 89 families obtained by random mating between 5–11 single plants. Single plants were selected from the highest biomass yielding F_2_ families, by visual merits and according to the synchronous heading time. After crossing, SYNs production followed the same protocol described for F_2_ families, involving pooling, multiplication of the seed in isolated plots, and testing in field trials.

Sequence data was produced by Genotyping-By-Sequencing (GBS) [49]. GBS uses methylation sensitive restriction enzymes (such as ApeKI) to target the low copy fraction of the genome, and can be used to estimate genome-wide allele frequency profiles in breeding populations [50]. Sampling and library preparation followed the protocol described by Byrne et al. [50], and Elshire et al. [49]. A total of 32 libraries were prepared, each of them containing up to 64 F_2_ families, and sequenced on multiple lanes of on an Illumina HiSeq2000 (single-end). After basic data filtering, the average number of reads per family was about 20 million. Data for each family was then aligned against a draft sequence assembly. 1,879,139 SNPs were identified, distributed across 30,285 scaffolds. Sequencing depth at a SNP ranged from 1 to 250 (upper limit) reads per family. SNP positions having more than 60 reads were discarded, as suspected to be originated from plastid genomes or from highly repetitive regions not captured in the draft assembly. No threshold was set in relation to the minimum number of reads. That could lead to a poor estimation of the allele frequencies and, consequently, to underestimation of allele substitution effect. However, it is possible to take account of this problems by using specific corrections, as showed by Ashraf et al. [51]. Markers were also filtered based on allele frequencies, removing SNPs with an estimated MAF lower than 0.02. After that, a total 1,447,122 markers were available for analyses. A further filtering was performed for GWAS and LD analyses (MAF > 0.05), leaving a total of 1,005,590 SNPs.

Phenotypic data were collected, within the standard breeding procedures of DLF. Families were sown during spring and scored during the following season. HD was assessed on family means by visual scoring, and defined as the day in which, two-thirds of the spike is visible on at least one plant in the plot or one third of the spike is visible in three plants in the plot. The character was expressed as ‘days after May 1^st^’. Data were available for a period of 11 years (between 2003 and 2013), and for two locations: Store Heddinge (South-Eastern Denmark) and Didbrook (Southern England). Fields were divided in trials, each consisting of randomized 24 sward plots, arranged in 2 sub-trials. Plot size was 1.5*10 m in Denmark and 0.5*4 m in England. Randomization was ensured within trials, but not always across trials. In some cases, especially in the oldest experiments, families were sorted according to the flowering time, or to the year of origin. That resulted in a certain degree of unbalance, within locations, between trials and PPs. A summary of the phenotypic data is displayed in Table 3, which shows the number of phenotyped families, along with the number of environments (location × year) where data were recorded, and some descriptive statistics (mean, standard deviation [SD], minimum, and maximum).

**Table 3:** Summary statistics for F_2_ and SYN families

	F_2_s	SYNs
N. phenotyped families	1757	89
N. locations	2	2
N. environments (location*year)	10	4
N. replicates	3.9	2.3
N. location per family	1.56	1.18
N. environments per family	1.98	1.18
Mean	25.9	31.5
SD	8.6	8.6
Min	3	13
Max	51	50

### Population Structure and LD

A Genomic relationship matrix (G-matrix) for all families was calculated from all SNP markers, after filtering for SNP depth and allele frequency (MAF > 0.02). Firstly, allele frequencies were arranged in a matrix **X**_(*i*×j)_, with *i* indexing marker, and *j* indexing family. The matrix was then centered by mean SNP frequencies $\left({\mathbf{M}}_{ij}={\mathbf{X}}_{ij}-{\overline{\mathbf{X}}}_i\right)$, where missing data were imputed with the average allele frequency, and used to compute **G**:

1 $$ \mathbf{G}=\mathbf{M}^{\prime}\mathbf{M}/\mathbf{K} $$

where **K** is a scaling parameter, corresponding to the sum of expected SNP variances as computed by Ashraf et al. [51], being 0.25 ${\displaystyle \sum_{i=1}^{\mathrm{N}}}{\overline{\mathbf{X}}}_i\left(1-{\overline{\mathbf{X}}}_i\right)$, with N equal to number of markers. Then, a PCA was performed on the G-matrix. The best number of clusters was determined by k-means clustering, using the R package ‘NbClust’ [52]. The probability, for each family, to belong to each cluster was computed with the R package ‘e1071’ [53]. LD within scaffolds was measured across all the F_2_ families on a set of 100 scaffolds larger than 20 kbp, randomly sampled across the whole genome. The LD was expressed as squared correlation between markers (r^2^). Corrections for both relatedness and for population structure were performed according to the method described by Mangin et al. [54].

### Statistical models and genetic parameters

Data were analyzed by linear mixed models, using the software DMU [55, 56]. The genomic information was implemented by using the G-matrix as variance covariance structure of the breeding values. Due to the not perfectly randomized design, the trial effect was included in the fixed part of the model [57]. Different models were tested on the F_2_ set and compared by *F*-test (for the fixed part) and Akaike test (for the random part). The models that showed the best fit to the data is reported below:

2 $$ \mathbf{y}=\mathbf{X}\mathbf{t}+{\mathbf{Z}}_{\mathbf{1}}\mathbf{i}+{\mathbf{Z}}_{\mathbf{2}}\mathbf{i}\mathbf{l}\mathbf{y}+{\mathbf{Z}}_{\mathbf{3}}\mathbf{p}+{\mathbf{Z}}_{\mathbf{4}}\mathbf{p}\mathbf{p}\mathbf{l}\mathbf{y}+\mathbf{e} $$

where **y** is the vector of phenotypes; **X** is the design matrix for the fixed factor; **t** is the vector of trial effects nested within location and year; **Z**_***i***_ are design matrices for random factors; **i** is a vector of breeding values ~ N(0, **Gσ**^**2**^_**i**_ ), where **G** is the G-matrix; **ily** is a vector of genotype × environment interactions ~ N(0, I**σ**^**2**^_**ily**_); **p** is a vector of the originating PPs ~ N(0, **Pσ**^**2**^_**p**_), with **P** being a genomic relationship matrix among PPs (P-matrix) built as described in the following paragraph; **pply** is the vector of interaction between PPs (which would mainly arise from dominance effects) nested within environments ~ N(0, I**σ**^**2**^_**pply**_); **e** is a vector of random residuals ~ N(0, I**σ**^**2**^_**e**_). Additional factors for breeding values and PPs, with identity matrices as variance-covariance structure were tested to check for presence of genetic effects not explained by G- and P- matrix. However, such effects turned out to be not significantly different from zero and were left out from the model. The same was for the interactions among PPs and between PPs and environments, and for the spatial effect within trials. Breeding values were calculated by summing the corresponding solutions for **i** and **p**:

3 $$ {\widehat{\mathbf{g}}}_j={\widehat{\mathbf{i}}}_j+{\widehat{\mathbf{p}}}_{j_1}+{\widehat{\mathbf{p}}}_{j_2} $$

where *j* indicates family and *j*_1_ and *j*_2_ indicates the parents population for family *j*.

Matrix **Z**_**3**_ was built to account for the presence of multiple PPs, as shown in Additional file 5: Figure S3 in the supplementary material. In each row, numbers indicate the expected probability, for each allele, to come from each PP. As each locus has two alleles, the numbers on each row sum up to two. **P** was computed based on the estimated frequencies of the PPs, following the same procedure that used to compute G-matrix. PPs frequencies were estimated for each SNP marker, using the following model:

4 $$ {\mathbf{f}}_{\boldsymbol{i}}={\boldsymbol{\upmu}}_{\boldsymbol{i}}+\mathbf{Z}{\mathbf{p}}_{\boldsymbol{i}}+{\mathbf{e}}_{\boldsymbol{i}} $$

where **f**_**i**_ is the vector of frequencies for marker ***i***; **μ**_**i**_ is the mean frequency for marker ***i***; **Z** is a matrix of random effect, accounting for the presence of multiple PPs, built as explained in Additional file 5: Figure S3; **p**_***i***_ is a vector of originating PPs ~ N(0, I**σ**^**2**^_**p**_). The estimated PPs frequency for a marker ***i*** was equal to:

5 $$ \mathbf{f}{\mathbf{p}}_{\boldsymbol{i}}={\boldsymbol{\upmu}}_{\boldsymbol{i}}+\mathbf{2}{\mathbf{p}}_{\boldsymbol{i}} $$

The model was based on the additive biallelic infinitesimal model described by Ashraf et al. [51], which was built on the following assumptions: (i) large number of individuals in PPs, F_1_ and F_2_ families; (ii) PPs in Hardy-Weinberg equilibrium; (iii) large number of families originated by each parent combination; (iv) parent plants chosen at random from the PPs; (v) absence of self-pollination; (vi) no intercross among F_1_ families; (vii) absence of selection between F_1_s and F_2_s; (viii) uniform variances across different factors. Here, the only difference in respect to the original model is represented by the relationship among PPs. That would cause inbreeding between the F_1_’s, [51] and lead to changes in frequencies and variances among PPs and F_2_s (described by P-matrix and by G-matrix respectively), and within F_2_s. The latter component can be ignored, as analyses are based on family means. The G-matrix also accounts for the increase in inbreeding within the F_2_ families. Variance components were estimated by restricted maximum likelihood method (REML), and can be interpreted as follows: **σ**^**2**^_**i**_ is the additive genetic variance among families, across environments; **σ**^**2**^_**ily**_ is the additive G × E variance; **σ**^**2**^_**p**_ is the variance among PPs across environments; **σ**^**2**^_**pply**_ is the variance of the G × E for dominance; **σ**^**2**^_**e**_ is the variance of residuals, which includes environmental effects within plots and measurement errors.

Across PPs, it was possible to compute three kinds of heritabilities for a single observation: (6) narrow sense heritability across environments; (7) narrow sense environment-specific heritability; (8) broad sense environment-specific heritability:

6 $$ {{\mathbf{h}}^{\mathbf{2}}}_{\mathbf{n}}=\left(\mathbf{G}{{\boldsymbol{\upsigma}}^{\mathbf{2}}}_{\mathbf{i}}+\mathbf{2}\mathbf{P}{{\boldsymbol{\upsigma}}^{\mathbf{2}}}_{\mathbf{p}}\right)/{{\boldsymbol{\upsigma}}^{\mathbf{2}}}_{\mathbf{P}} $$

7 $$ {{\mathbf{h}}^{\mathbf{2}}}_{\mathbf{ne}}=\left(\mathbf{G}{{\boldsymbol{\upsigma}}^{\mathbf{2}}}_{\mathbf{i}}+\mathbf{2}\mathbf{P}{{\boldsymbol{\upsigma}}^{\mathbf{2}}}_{\mathbf{p}}+{{\boldsymbol{\upsigma}}^{\mathbf{2}}}_{\mathbf{i}\mathbf{ly}}\right)/{{\boldsymbol{\upsigma}}^{\mathbf{2}}}_{\mathbf{P}} $$

8 $$ {{\mathbf{h}}^{\mathbf{2}}}_{\mathbf{be}}=\left(\mathbf{G}{{\boldsymbol{\upsigma}}^{\mathbf{2}}}_{\mathbf{i}}+\mathbf{2}\mathbf{P}{{\boldsymbol{\upsigma}}^{\mathbf{2}}}_{\mathbf{P}}+{{\boldsymbol{\upsigma}}^{\mathbf{2}}}_{\mathbf{i}\mathbf{ly}}+{{\boldsymbol{\upsigma}}^{\mathbf{2}}}_{\mathbf{pply}}\right)/{{\boldsymbol{\upsigma}}^{\mathbf{2}}}_{\mathbf{P}} $$

where the component **σ**^**2**^_**p**_ was added twice, as each F_2_ family was originated from two PPs, and where **σ**^**2**^_**P**_ is the phenotypic variance, calculated as:

9 $$ {{\boldsymbol{\upsigma}}^{\mathbf{2}}}_{\mathbf{P}}=\mathbf{G}{{\boldsymbol{\upsigma}}^{\mathbf{2}}}_{\mathbf{i}}+{{\boldsymbol{\upsigma}}^{\mathbf{2}}}_{\mathbf{i}\mathbf{ly}}+\mathbf{2}\mathbf{P}{{\boldsymbol{\upsigma}}^{\mathbf{2}}}_{\mathbf{p}}+{{\boldsymbol{\upsigma}}^{\mathbf{2}}}_{\mathbf{p}\mathbf{ply}}+{{\boldsymbol{\upsigma}}^{\mathbf{2}}}_{\mathbf{e}}. $$

### Genome Wide Association and Genomic Prediction

GWAS analysis was performed by using the software GAPIT [58]. Correction for relatedness was ensured by the use of G-matrix as kinship matrix. A further correction for population structures was carried out by adding the main four PCs to the model. The optimal number of PCs was determined by GAPIT through Bayesian Information Criterion. The model used for GWAS was the following:

10 $$ \widehat{\mathbf{g}}={\mathbf{X}}_{\mathbf{1}}{\boldsymbol{\upalpha}}_{\mathbf{0}\boldsymbol{i}}+{\mathbf{X}}_{\mathbf{2}}\mathbf{p}\mathbf{c}+\mathbf{Z}\mathbf{i}+\mathbf{e} $$

where ĝ is a vector of breeding values, calculated from the model shown in equation (3), but assuming all variance covariance matrices to be identity matrices; **X**_**i**_ and **Z** are design matrices for fixed and random effects respectively; **α**_**0*i***_ is the allele substitution effect for locus *i*; **pc** is the vector for PCs effects; **i** is the vector of breeding values with G-matrix as variance-covariance structure distributed as N(0, **Gσ**^**2**^_**i**_); **e** is a vector of random residuals, distributed as N(0, I**σ**^**2**^_**e**_). Missing genotypes, for each marker, were imputed with the average allele frequencies across families, as was done for computing G-matrix. The significance of each marker effect was evaluated using t-test after Bonferroni correction and FDR [59], using a cut off level of 0.05. In case there were two or more significant markers in the same scaffold, an LD analyses was performed within the scaffold. When SNPs were in LD (r^2^ > 0.10), only the marker with the lowest P-values was regarded as significant. Allele substitution effects were corrected for low sequencing depth [51], using the following formula:

11 $$ {\alpha}_i={\alpha}_{0i}\ast \left(1+3/{\mathrm{D}}_i\right) $$

where α_0_ is the allele substitution effect as estimated from GWAS, α is the corrected allele substitution effect, D is the average sequencing depth across families, and *i* refers to a given locus.

As the allele frequencies are expressed on family means, the genetic variance would be half of the variance between individuals [51], and the variance explained by each marker should be computed with the following formula:

12 $$ {{\boldsymbol{\upsigma}}^{\mathbf{2}}}_{\mathbf{g}\boldsymbol{i}}={\mathbf{p}}_i\left(1-{\mathbf{p}}_i\right){\upalpha_i}^2 $$

where p is the MAF, α is equal to the allele substitution effect, and *i* refers to a given allele. For all the SNPs that were declared significant in at least one of the tests, **σ**^**2**^_**g*i***_ was calculated both within the F_2_ families, and using the MAF of the SYN families. Then, a single marker regression was performed in the SYN families, in order to check the association between marker effects in the two sets. Later, all SNPs were ordered based on their P-values, and then used to estimate the GEBVs of the SYN families:

13 $$ \widehat{\mathbf{g}}={\displaystyle \underset{i=1}{\overset{\mathrm{M}}{\varSigma }}}{\upalpha}_i\ast {\mathbf{p}}_i $$

in this equation, **ĝ** is the vector of GEBVs, M is equal to the number of significant markers, α_*i*_ is the allele substitution effect for marker ***i***, and **p**_***i***_ is the MAF at marker ***i***. The calculation was performed multiple times, assuming different values for M (from 1 to 50 markers).

GP studies were carried out by GBLUP [60, 61], using CV within different F_2_ sets: (i) all F_2_ families; (ii) different clusters of F_2_ families, previously identified during the PCA; (iii) reduced sets of randomly chosen F_2_s, differing for size of the training populations. Within each set, CV was performed according to two different schemes testing different hypothesis: (a) *k*-*fold* (k = 100) tests predictions in case of presence of related individuals in the training and in the validation set, leaving out families in random order and estimates their breeding values; (b) *pp-fold* tests predictions in case of absence of related individuals in the training and in the validation set, estimating all the families originated by a certain PPs combination, after having left out everything that had at least a PP in common. As *pp-fold* implied a greater reduction in terms of training population compared to *k-fold*, a *pp-like* strategy was also tested, in order to ensure the same training population size is used in both schemes. This strategy exactly replicated the cross-validation scheme used in *pp-fold*, but leaving out random families instead. Analyses on set (iii) were repeated ten times, each time using a different set of randomly chosen F_2_s, and the average predictive abilities and bias were calculated. Then, CVs were performed across clusters and, finally, all the F_2_s were used to predict the breeding values of the SYN families.

Accuracy is defined as the correlation between true breeding values and GEBVs (**ρ**_**g,ĝ**_). In this case, its value is not known, but can easily be computed by using the following equation [62]:

14 $$ {\boldsymbol{\uprho}}_{\mathbf{g},\widehat{\mathbf{g}}}={\boldsymbol{\uprho}}_{\overline{\mathbf{y}}\mathbf{f},\widehat{\mathbf{g}}}/{\boldsymbol{\uprho}}_{\overline{\mathbf{y}}\mathbf{f},\mathbf{g}} $$

where the nominator is the true correlation between GEBVs with the average phenotypes corrected for the fixed effect (**y**_**f**_), defined as predictive ability, and the denominator represents the expected correlation between GEBVs and ${\overline{\mathbf{y}}}_{\mathbf{f}}$. Such a formula gives an estimation of the correlation between **g** and **ĝ**, which is not guaranteed to fall within the theoretically defined range of the parameters. The expected correlation between GEBVs and ${\overline{\mathbf{y}}}_{\mathbf{f}}$ can be calculated with the following equation [63]:

15 $$ {\boldsymbol{\uprho}}_{\overline{\mathbf{y}}\mathbf{f},\mathbf{g}}={\boldsymbol{\upsigma}}_{\mathbf{g}}\ast {\left({{\boldsymbol{\upsigma}}^{\mathbf{2}}}_{\mathbf{g}}+{{\boldsymbol{\upsigma}}^{\mathbf{2}}}_{\mathbf{e}}/\mathbf{n}\right)}^{-\left(1/2\right)} $$

where **σ**^**2**^_**g**_ is the genomic variance, and **n** number of replicates. That is equivalent to the square root of the heritability based on family means (based on several observations), and represents the upper limit for the prediction accuracy. However, this formula refers to a very simplified model with only genomic and residual variances. In the present paper, the equation needs to account for the other random components:

16 $$ {\boldsymbol{\uprho}}_{\overline{\mathbf{y}}\mathbf{f},\mathbf{g}}=\sqrt{\mathbf{G}{{\boldsymbol{\upsigma}}^{\mathbf{2}}}_{\mathbf{i}}+\mathbf{2}\mathbf{P}{{\boldsymbol{\upsigma}}^{\mathbf{2}}}_{\mathbf{p}}}\ast {\left(\mathbf{G}{{\boldsymbol{\upsigma}}^{\mathbf{2}}}_{\mathbf{i}}+\mathbf{2}\mathbf{P}{{\boldsymbol{\upsigma}}^{\mathbf{2}}}_{\mathbf{p}}+{{\boldsymbol{\upsigma}}^{\mathbf{2}}}_{\mathbf{i}\mathbf{ly}}/{\mathbf{n}}_{\mathbf{i}\mathbf{ly}}+{{\boldsymbol{\upsigma}}^{\mathbf{2}}}_{\mathbf{p}\mathbf{ply}}/{\mathbf{n}}_{\mathbf{p}\mathbf{ply}}+{{\boldsymbol{\upsigma}}^{\mathbf{2}}}_{\mathbf{e}}/\mathbf{n}\right)}^{-\left(1/2\right)} $$

As different families were replicated a different number of times, **n** is the average number of replicates across all fields (n_plots_/n_families_); **n**_**ily**_ is the average number of environments per each family (n_ily_/n_families_) and **n**_**pply**_ is the average number of environments per each PP (n_PPly_/n_PP_). When predictions were performed on the same dataset, comparison between predictive abilities from different models was performed with Hotelling-Williams test [64], using the R script developed by Christensen et al. [65]. The bias of the predictions was investigated by regressing ${\overline{\mathbf{y}}}_{\mathbf{f}}$ on the breeding value estimates:

17 $$ {\overline{\mathbf{y}}}_{\mathbf{f}}=b\widehat{\mathbf{g}}+c;\kern1em b={\boldsymbol{\upsigma}}_{\overline{\mathbf{y}}\mathbf{f},\widehat{\mathbf{g}}}/{{\boldsymbol{\upsigma}}^{\mathbf{2}}}_{\widehat{\mathbf{g}}} $$

where ${\overline{\mathbf{y}}}_{\mathbf{f}}$ is the vector of corrected and average phenotypes not included in computing GEBVs, and **ĝ** is the vector of GEBVs from the CV procedure. Absence of bias will result in a regression coefficient (*b*) of 1. A significant deviation from 1 indicates bias in the estimation of the GEBVs’ variance.

## Additional files

Additional file 1: Figure S1. PCA scree plot. (BMP 13 kb) Additional file 2:
**Genes predicted in the scaffolds harboring the significant SNPs.** (XLSX 14 kb) Additional file 3: Table S1. Scaffolds with significant SNPs. (DOCX 14 kb) Additional file 4: Figure S2. A dot plot of the sequence alignment of the genomic scaffold (first sequence) that contains the SNP significantly associated with heading date, and proximal to *CO*, against the 29Kb region (SEQ2) sequenced in the study of Skøt et al., [29]. (BMP 2159 kb) Additional file 5: Figure S3. Construction of the design matrices for PPs. Legend: F_2_*j*_, SYN_*j*_ = families; PP_*i*_ = parent populations. (BMP 288 kb)

## Abbreviations

bp: base pairs
CV: cross-validation
FDR: false discovery rate
G × E: genotype by environment
GBLUP: genomic best liner unbiased prediction
GBS: genotype by sequencing
GEBV: genomic estimated breeding value
GP: genomic prediction
GS: genomic selection
GWAS: Genome Wide Association Analysis
HD: heading date
LD: linkage disequilibrium
LG: linkage group
MAF: minor allele frequency
MAS: marker assisted selection
PP: Parent population
QTL: quantitative trait locus
SYN: synthetic familiy
