# Supplementary figures and images for: Genomic dissection and prediction of heading date in perennial ryegrass
Source: BMC Genomics. 2015 Nov 11;16:921. doi: 10.1186/s12864-015-2163-3 (PMC4642674; doi:10.1186/s12864-015-2163-3)

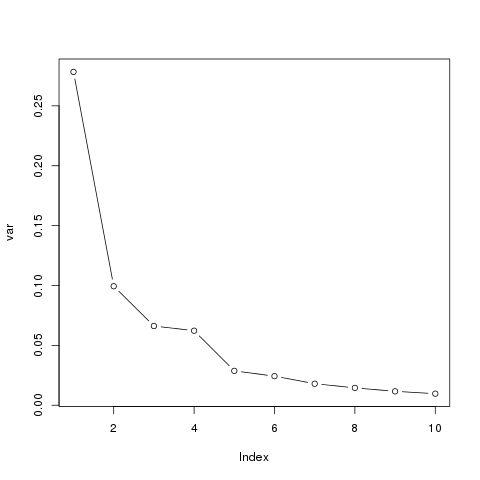

Supplement: Additional file 1: Figure S1. — PCA scree plot. (BMP 13 kb) [file 12864_2015_2163_MOESM1_ESM.bmp]

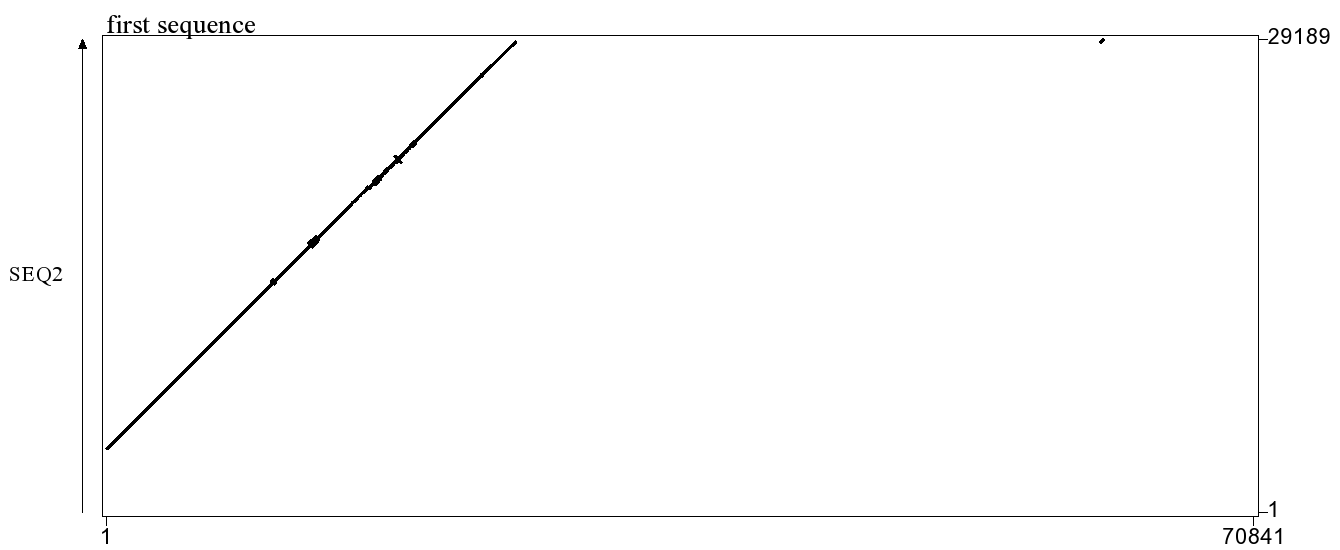

Supplement: Additional file 4: Figure S2. — A dot plot of the sequence alignment of the genomic scaffold (first sequence) that contains the SNP significantly associated with heading date, and proximal to CO, against the 29Kb region (SEQ2) sequenced in the study of Skøt et al., [29]. (BMP 2159 kb) [file 12864_2015_2163_MOESM4_ESM.bmp]

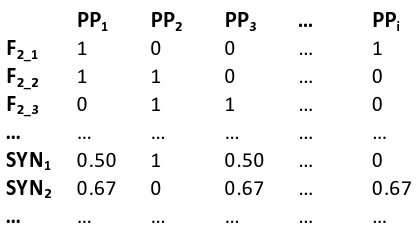

Supplement: Additional file 5: Figure S3. — Construction of the design matrices for PPs. Legend: F2_j, SYNj = families; PPi = parent populations. (BMP 288 kb) [file 12864_2015_2163_MOESM5_ESM.bmp]
