# Supplementary material for: Genomic dissection and prediction of heading date in perennial ryegrass
Source: BMC Genomics. 2015 Nov 11;16:921. doi: 10.1186/s12864-015-2163-3 (PMC4642674; doi:10.1186/s12864-015-2163-3)
Supplement: Additional file 3: Table S1. — Scaffolds with significant SNPs. (DOCX 14 kb) [file 12864_2015_2163_MOESM3_ESM.docx]

| **Table S1. Scaffolds with significant SNPs.** | | | |
| --- | --- | --- | --- |
| **Scaffold** | **Scaffold Size (bp)** | **No. predicted genes** |  |
| Scaffold_3546 | 89,535 | 1 |  |
| Scaffold_18961 | 9,347 | 1 |  |
| Scaffold_6570 | 55,739 | 5 |  |
| Scaffold_22974 | 5,351 | 0 |  |
| Scaffold_1379 | 114,493 | 6 |  |
| Scaffold_18588 | 9,827 | 1 |  |
| Scaffold_9291 | 29,242 | 0 |  |
| Scaffold_9679 | 38,076 | 2 |  |
| Scaffold_2801 | 94,162 | 4 |  |
| Scaffold_5059 | 70,841 | 3 |  |
| Scaffold_3169 | 87,980 | 3 |  |
| Scaffold_21110 | 6,649 | 0 |  |
| Scaffold_3586 | 84,513 | 6 |  |
| Scaffold_3395 | 85,176 | 2 |  |
